# Low-energy shock waves improve the bacterial detection of *Staphylococcus aureus* biofilms on polyethylene

**DOI:** 10.1038/s41598-025-16834-4

**Published:** 2025-08-26

**Authors:** Sabrina Böhle, Victoria Horbert, Sebastian Rohe, Chris Lindemann, Eric Röhner, Georg Matziolis

**Affiliations:** 1 Orthopaedics University Hospital Jena, Campus Eisenberg, Waldkliniken Eisenberg, Friedrich-Schiller-University, Klosterlausnitzer Straße 81, 07607 Eisenberg, Germany; 2 Orthopaedic Department, Heinrich-Braun-Klinikum, 08060 Zwickau, Germany

**Keywords:** *Staphylococcus aureus*, Biofilm, Extracorporeal shock waves, Low-energy shock waves, Periprosthetic infection, Polyethylene, Biofilms, Diagnosis, Experimental models of disease

## Abstract

With the increasing number of total joint arthroplasties and the associated increase in periprosthetic infections, the further development of non-invasive examination methods to improve bacterial detection is becoming increasingly important. This is particularly important in the case of biofilm-forming bacteria, where false-negative results from joint puncture can lead to a delay in optimal therapy, as the number of planktonic bacteria in the punctate can be low. Extracorporeal shock wave therapy, originally used in the treatment of urolithiasis, has demonstrated promising energy-dependent biofilm-disrupting and even antimicrobial properties against *Staphylococcus aureus*. High-energy shock waves have been shown to be effective in several studies, but they are often painful and not suitable for all patients. Utilizing shock waves could enhance pathogen detection rates and potentially enable the early initiation of targeted therapy. This study therefore investigates whether low-energy shock waves are suitable for removing bacteria from a *Staphylococcus aureus* biofilm on polyethylene. The aim of this study is to evaluate the applicability of this method to improve the diagnostic accuracy of periprosthetic infections. In an in vitro model, *Staphylococcus aureus* biofilms were cultured on polyethylene patellas for 48 h. Biofilm disruption by low-energy shock waves was tested using a ReflecTron hmt device, with shock waves applied in a range of 0–1800 impulses. Colony-forming units (CFU) and XTT assays (to quantify cell viability) were measured. Shock wave treatment with an energy of 0.13 mJ/mm^2^ proved to be effective in removing bacteria from *Staphylococcus aureus* biofilms on polyethylene surfaces. A significant increase in CFU within the surrounding solution was observed after just 100 impulses (*p* = 0.018), and continued to increase until approximately 900 impulses. A linear correlation was identified between the logarithm of the shock wave impulses and both the CFU (r = 0.971, *p* < 0.001) and the XTT activity (r = 0.94, *p* < 0.001). This finding suggests that low-energy shock waves detach living bacteria from the biofilm. Consequently, they highlight the potential of low-energy shock waves to effectively disrupt biofilms without compromising bacterial viability, reinforcing their potential diagnostic and therapeutic applications. Low-energy shock waves disrupt *Staphylococcus aureus* biofilms on polyethylene surfaces in vitro, dislodging bacteria from the biofilm. However, further in vivo studies are required in order to assess the potential of this method for clinical applications. Such studies could determine whether shock waves can enhance periprosthetic infection diagnosis in vivo and facilitate implant-preserving therapies for mature biofilms.

## Introduction

With the increasing number of joint replacements being performed^1^, periprosthetic infections are also expected to increase^2^. Successful medical therapy of periprosthetic joint infection (PPI) begins with an accurate microbiologic diagnosis to enable the most effective antimicrobial therapy. The focus is on bacteria that can colonize medical implants and lead to persistent PPI^3^, such as *Staphylococcus aureus*, which is the most important pathogen in orthopedic infections^4,5^. Once microorganisms come into contact with the implant surface, they colonize it and form a biofilm^6^. This biofilm significantly reduces susceptibility to antibiotics^7^ and also affects diagnosic accuracy, thus impacting the success of infection treatment. This is due to the fact that most organisms are concentrated in a biofilm directly on the implant, while only a few planktonic bacteria are present in the joint fluid^6,8^. As a result, the gold-standard joint puncture may fail to detect pathogens^6,8^. However, several strategies exist to identify these pathogens, such as sonication^6,9^. In sonication low frequency ultrasound waves are used to dislodge biofilm microorganisms from the surface of the implants^10^. The culture of sonication fluid is more sensitive than standard periprosthetic tissue culture and also provides good results after previous antibiotic therapy^9,11^. However, these tests are invasive and require surgery for sample collection. Starting with the treatment of urolithiasis^12^, the non-invasive extracorporeal shock wave therapy has now become an integral part of the treatment of non-union, delayed healing, osteochondrosis dissecans, plantar fasciitis, Achilles tendinopathy, and epicondylitis radialis, among others^13–15^. In addition, high-energy extracorporeal shock waves (ESW) have a bactericidal effect on *Staphylococcus aureus*^16–18^. Recent studies have demonstrated the potential of high energy extracorporeal shock wave therapy in disrupting bacterial biofilms and enhancing antibiotic efficacy. Milstrey et al. showed that high-energy focused ESW significantly reduced bacterial colonization on titanium discs in vitro. The combination of shock waves with antibiotics such as rifampicin further improved the antimicrobial effect^19^. Similarly, Gnanadhas et al. found that shock wave therapy effectively disrupted biofilms of various bacterial species, restoring their susceptibility to antibiotics in both in vitro and in vivo models^20^. These findings indicate that ESW could serve as a promising alternative or adjunctive therapy for PPI. Qi et al. further demonstrated that the combination of high-energy extracorporeal shock waves with gentamicin significantly increased the efficacy against *Staphylococcus aureus* biofilms, both in vitro and in vivo, supporting the role of ESW as an adjunctive therapy for infections^21^.

Since most studies have investigated high-energy shock waves, which are often painful and may not be well tolerated by sensitive patients, low-energy shock waves may be a good alternative. In addition, high energy ESW may damage the cement mantle and result in early implant loosening or third-body wear from cement particles when applied to cemented implants. Wanner et al. investigated the effect of low-energy shock waves on *staphylococcal* biofilms on stainless steel washers and found that this approach significantly enhanced the susceptibility of biofilms to antimicrobial agents in vitro^22^. The purpose of this in vitro study is to determine the number of low-energy shock waves that can disrupt a *Staphylococcus aureus* biofilm on polyethylene.

## Materials and methods

### Bacterial biofilm

**Fig. 1:**
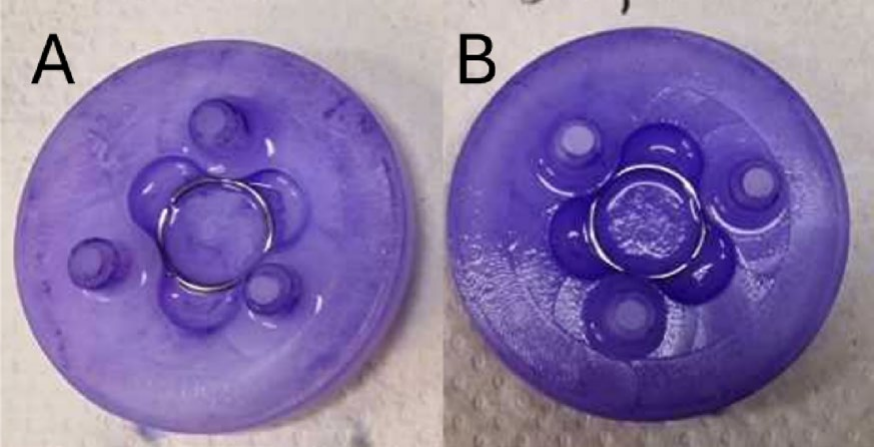
Visualization of *Staphylococcus aureus* biofilm with crystal violet dye (**A**) control with discrete staining of the polyethylene, (**B**) patella with biofilm).

*Staphylococcus aureus* strain DM 346 was freshly inoculated into CASO broth (Carl Roth GmbH & Co. KG, Karlsruhe, Germany) and incubated overnight at 37 °C for 24 h. The medium was then replaced and a polyethylene patella was added. The patella was incubated for 48 h in daily refreshed medium to form a biofilm. One sample patella was stained with crystal violet dye to visualize the bacterial biofilm (Fig. 1).

### Experiments with extracorporeal shock waves

**Fig. 2:**
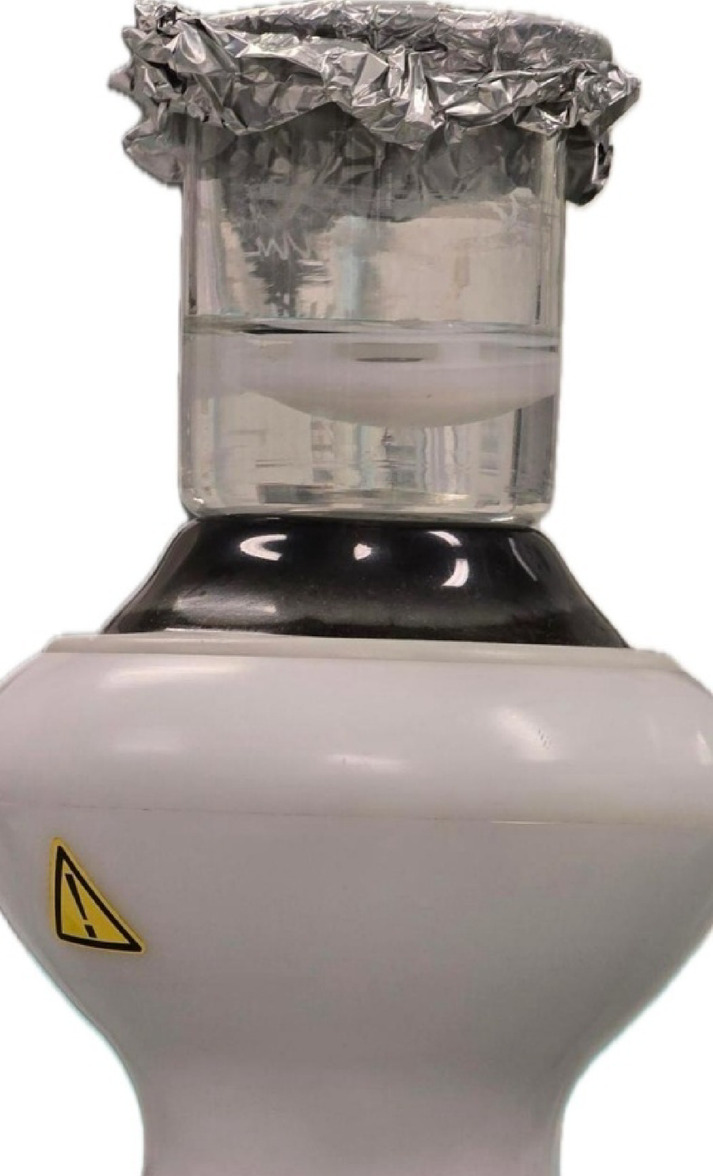
Set-up for shockwave application to the patella (Bottom: shockwave device; Top: beaker containing 20 ml of PBS and the patella).

The patella was then removed from the medium, rinsed five times with Phosphate Buffered Saline (PBS) to remove loosely adherent bacteria, and placed in a beaker containing 20 ml of PBS. Shock waves of energy level E9 (0.13 mJ/mm^2^) were applied to the patella in the PBS solution using a ReflecTron device (HMT AG (Kreuzlingen, Switzerland). The beaker itself was directly positioned over the shock wave applicator probe with no medium or distance between. The applicator was in immediate contact with the base of the beaker, allowing vertical transmission of shock waves to the patella through the PBS solution. An image of the setup is shown in Fig. 2. The shock waves were administered in increments of 50 impulses from 0 to 300 impulses and then in increments of 300 impulses to a total of 1800 impulses. There were no temperature fluctuations during the application, the tests were carried out at room temperature. The first sample of 202 µl was taken without prior pulsing. Afterwards, between each series of impulses, 202 µl of PBS solution was removed as samples. Moreover, both a positive sample (direct swab from the biofilm) and a negative sample (PBS) were used for the respective tests.

### CFU determination

100 µl of a 1:500 dilution, a positive control (medium containing bacteria to form a biofilm) and a negative control (medium) were plated on agar plates and incubated at 37 °C for 48 h. Colony-forming units (CFU/ml) were determined by enumeration. Data were obtained from four independent experiments, with two replicates per condition in each experiment.

### XTT ELISA detection of viable cells

The XTT assay was used to spectrophotometrically quantify cell growth and viability. This analysis is based on the cleavage of the tetrazolium salt XTT to a formazan dye by metabolically active cells. This conversion occurs only in viable cells. An increase in total XTT activity is directly proportional to an increase in the number of viable cells. Samples were plated twice each in 96-well plates, as well as negative control (PBS) and positive control (2% Triton X-100). After a 2-h incubation the samples were quantified by measuring the absorbance at a wavelength of 450–500 nm using an ELISA plate reader (FLUOstar OPTIMA, Microplate Reader, BMG LABTECH, Ortenberg, Germany). Data were obtained from three independent experiments with two replicates per condition per experiment. XTT results were interpreted qualitatively in parallel with CFU data to assess bacterial detachment and viability.

### Statistical analysis

The statistical evaluation was performed using SPSS Statistics Version 24 software for Windows (IBM, Armonk, USA). Data are presented as means ± standard deviation. Student’s paired *t* tests were used to analyze between the group without shock and each individual group afterwards. Correlations were evaluated using the Pearson test. A *p* value of < 0.05 was considered to be significant.

## Results

**Fig. 3:**
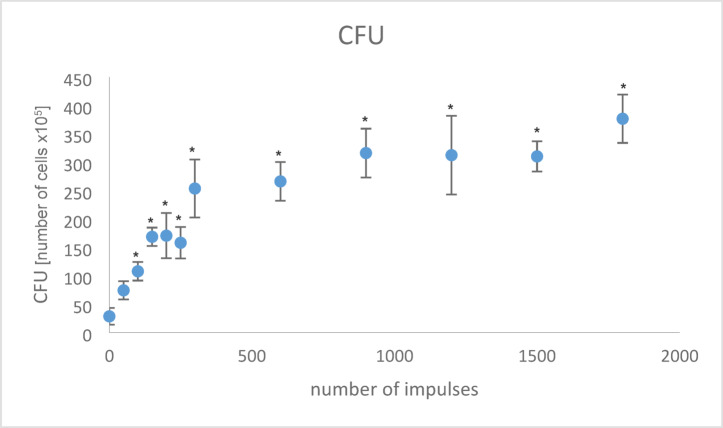
Number of colony-forming units (CFU) depending on shock wave impulses; *p < 0.05 to 0 impulses.

This in vitro model was used to investigate the optimal number of shock waves required to disrupt the biofilm of *Staphylococcus aureus* on polyethylene. Biofilm formation was optically detected using the crystal violet dye method, but not quantitatively analyzed. Evaluation of the CFU count (Fig. 3) showed a steep increase up to 150 and 300 impulses respectively, with a slight plateau thereafter. Interestingly, there was a significant increase after 100 impulses compared to 0 impulses (*p* = 0.018). The number of CFUs continued to rise to about 900 impulses, after which only minor fluctuations occurred without significant differences. In addition, no bacteria were detected in the negative sample with PBS. The positive sample showed complete growth of bacteria on the agar plate which could not be counted.

**Fig. 4:**
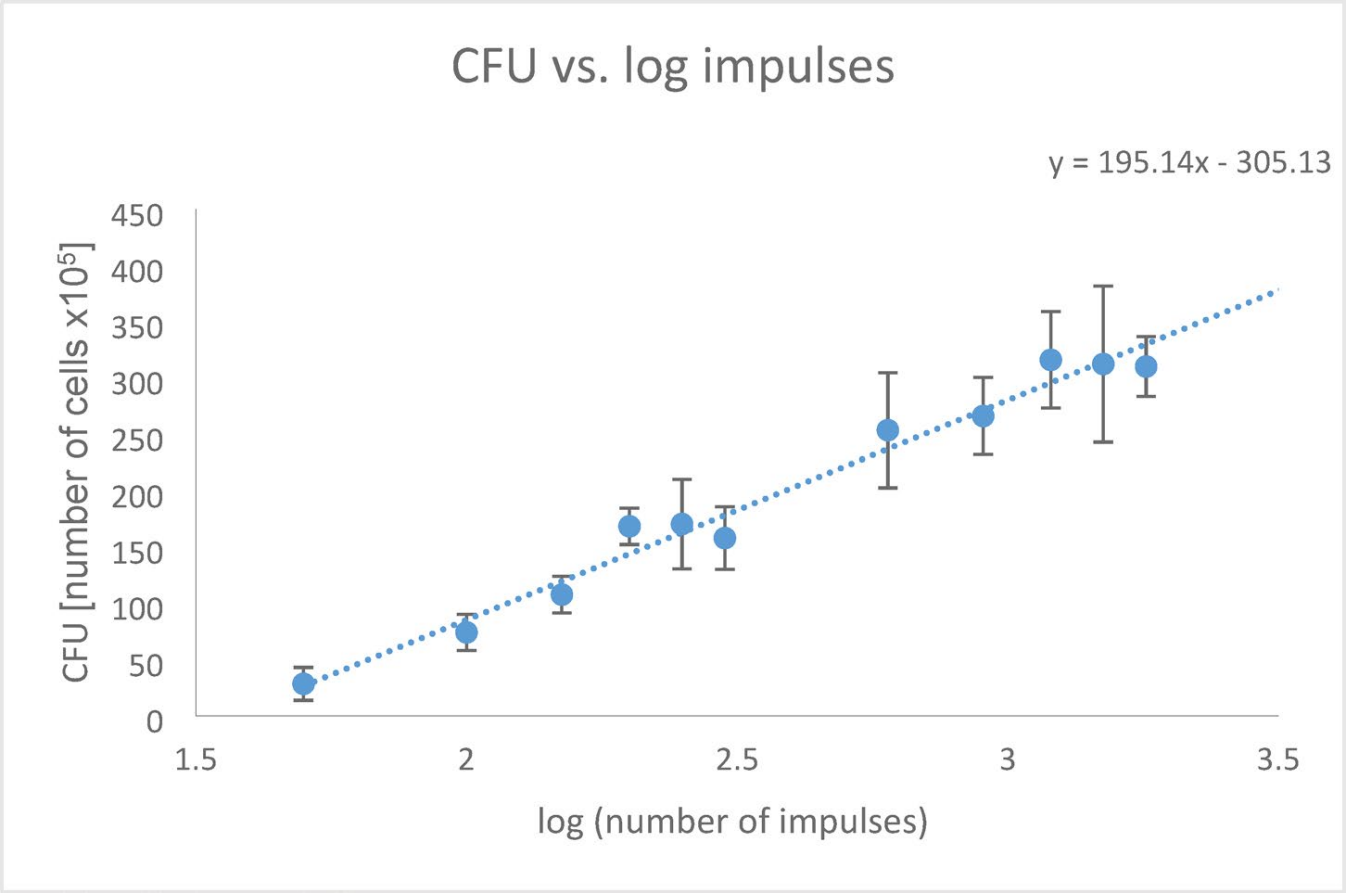
Number of CFU in relation to the logarithm of the shock wave impulses; Pearson correlation coefficient r = 0.971, *p* < 0.001.

Considering the logarithm of the respective shock wave impulses, a linear correlation with the number of CFU was observed (Fig. 4, y = 195.14x − 305.13). The Pearson correlation coefficient indicated a strong correlation (r = 0.971, *p* < 0.001).

**Fig. 5:**
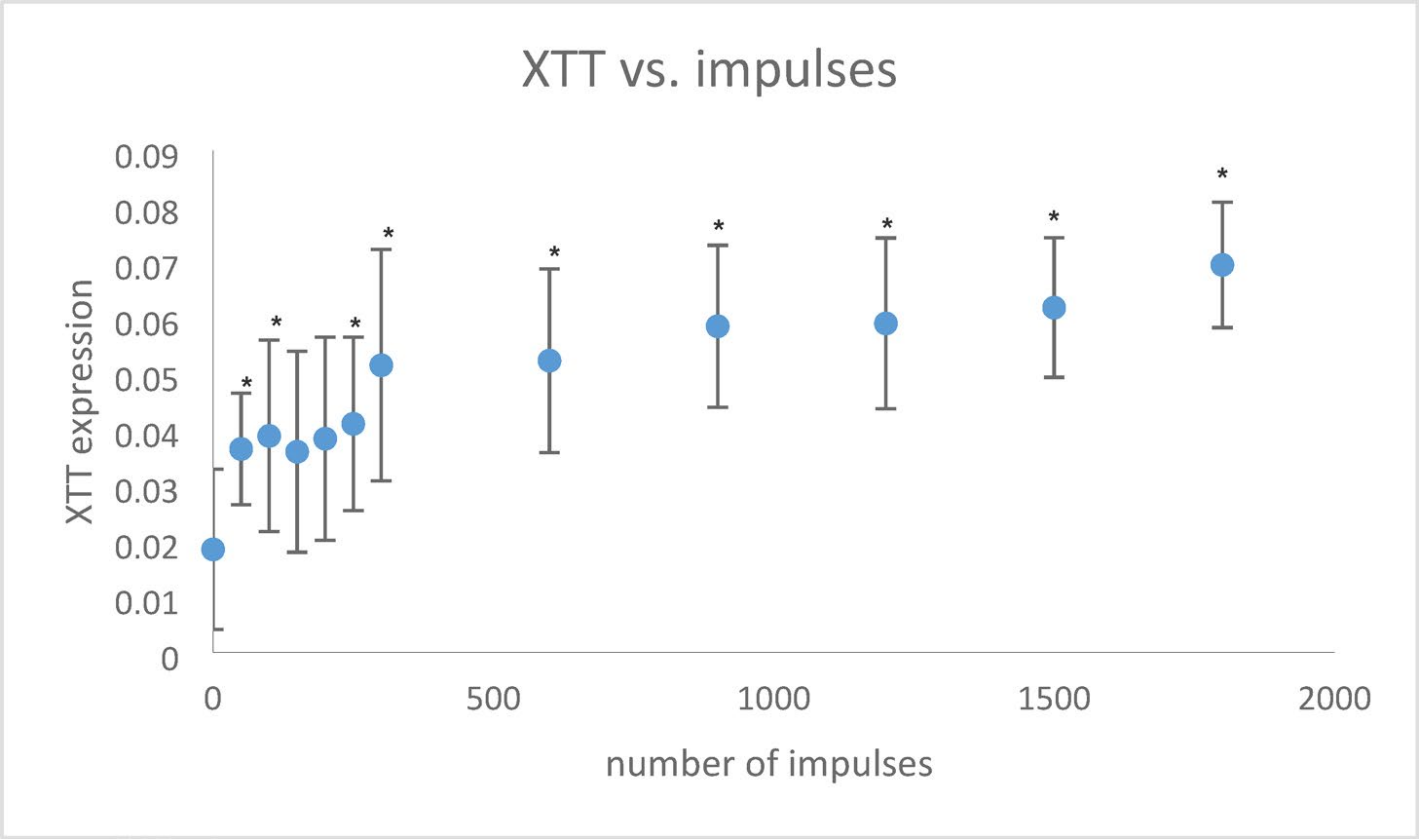
XTT expression depending on shock wave impulses; *p < 0.05 to 0 impulses.

Figure 5 illustrates that XTT expression, reflecting the number of viable cells in the medium, increased with the number of shock wave impulses. A significant rise in XTT values was detected after 50 impulses compared to 0 impulses (*p* = 0.041). Thereafter, the XTT expression increases only slightly up to 300 impulses, after which there were only minimal changes up to 900 impulses. The positive sample (2% Triton X-100) gave a value of 1.177 ± 0,032.

**Fig. 6:**
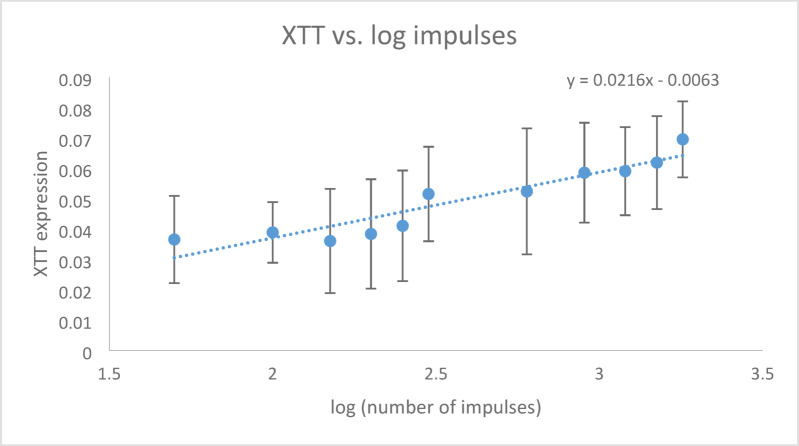
XTT expression as a function of the logarithm of the shock wave impulses; Pearson correlation coefficient r = 0.94, *p* < 0.001.

When XTT expression was plotted against the logarithm of shock wave impulses, it followed a linear function: y = 0.0216x − 0.0063 (Fig. 6). The Pearson correlation coefficient showed a high correlation (r = 0.94, *p* < 0.001).

**Fig. 7:**
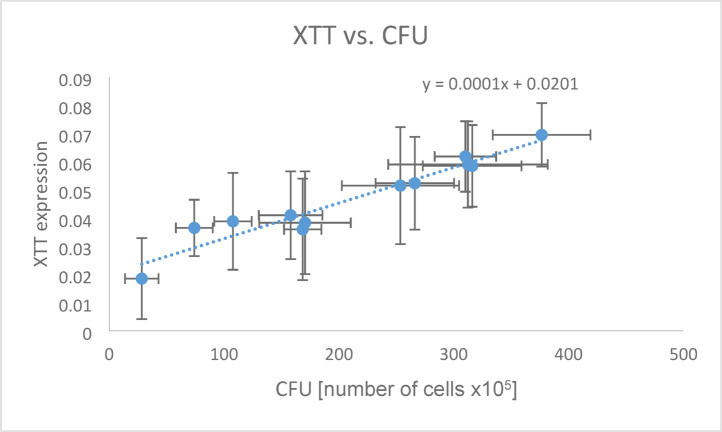
Ratio of XTT expression to number of CFU detected at shock wave application; Pearson correlation coefficient r = 0.965, *p* < 0.001.

Comparing CFU counts with XTT expression revealed an approximately linear relationship, described by the function y = 0.0001x + 0.0201 (Fig. 7). The Pearson correlation coefficient shows a very good correlation (r = 0.965; *p* < 0.001).

## Discussion

The results of this in vitro study show that even low-energy levels of extracorporeal shock waves (ESW) significantly affect the structure of *Staphylococcus aureus* biofilms on polyethylene. In particular, it was found that even 100 shock wave impulses caused a significant increase in colony-forming units (CFU) in the PBS solution compared to the sample without shock wave treatment. This early response suggests that low-energy ESW could be a potential method to disrupt the biofilm structure and release bacteria into the surrounding solution. The observed stagnation in the detection of bacteria as a surrogate parameter for biofilm disruption after approximately 300 impulses could be due to a saturation effect, where additional impulses do not further enhance the effectiveness of the shock waves.

The linear correlation between the logarithmic number of low-energy shock wave impulses and the number of CFU and XTT expression shows that viable bacteria are released from the biofilm in a impulse-dependent manner. In particular, the significant increase in XTT expression after only 50 shock wave impulses is remarkable. This indicates that even a small number of low-energy shock waves is sufficient to induce a measurable effect on the bacterial activity of the biofilm on polyethylene.

This confirms the hypothesis that the shock waves are directly related to the disruption of the structure of a biofilm. However, most studies that have investigated the bactericidal effect of shock wave therapy have used high-energy shock waves and bacterial suspensions rather than biofilms^16–18^. These observations are consistent with studies such as that of Qi et al., who demonstrated a synergistic effect of high-energy shock waves (0.96 mJ/mm^2^, 4000 impulses) in combination with gentamicin on the control of bacterial *Staphylococcus aureus* biofilms in vitro and in vivo^21^. Nevertheless, there are also controversial discussions on the synergism of shock waves and gentamicin. Horn et al. reported that the minimum inhibitory concentration against *Staphylococcus aureus* was not significantly affected by ESW application (0.59–0.96 mJ/mm^2^)^23^.

However, only a limited number of studies have investigated low-energy shock wave therapies in relation to biofilms. The study by Wanner et al. examined the effects of low-energy shock waves (0.16 mJ/mm^2^, 500 impulses) on the susceptibility of *Staphylococcus aureus* and *Staphylococcus epidermidis* biofilms to antimicrobial agents to assess a potential synergistic effect between ESW and antibiotics. The study demonstrated that ESW can increase the permeability of the biofilm matrix and thus increase the effectiveness of antimicrobial substances^22^. In contrast, the present study highlights the biofilm-disrupting effect of low-energy shock waves (0.13 mJ/mm^2^, up to 1800 impulses) on *Staphylococcus aureus* biofilms on polyethylene surfaces, without the use of antibiotics, to characterize the relationship between the number of impulses and the biofilm-disrupting effects more precisely. This approach enables a clearer understanding of the mechanical and structural effects of shock waves on biofilm architecture.

Our findings indicate that significant biofilm detachment can already occur after just 100 impulses, emphasizing the high sensitivity of early-stage biofilm structures to mechanical disruption. In contrast, Wanner et al. observed that shock wave treatment alone of *Staphylococcus aureus* and *Staphylococcus epidermidis* biofilms did not result in a significant reduction in the number of bacteria directly on the stainless steel surface (*p* = 0.08; *p* = 0.06). One potential explanation for this discrepancy could be the differences in biofilm growth times. While our biofilm matured within 48 h, a growth time of up to 72 h was selected in Wanner’s study^22^. This suggests that more mature biofilms may exhibit higher resistance to shock wave treatments.

Further methodological discrepancies exist between the studies: Wanner et al. used stainless steel washers as surface, whereas we chose polyethylene, a material frequently used in endoprosthetic inlays, to achieve a more authentic simulation of clinical conditions. Importantly, our use of polyethylene surfaces not only enhances clinical relevance but may also affect the shock wave transmission and energy coupling at the biofilm interface, thereby influencing the mechanical disruption achieved. The energy flux density used in this study (0.13 mJ/mm^2^) is classified as “low-energy” ESWT. According to Rompe et al., low-energy ESWT is defined as having an energy flux density below 0.2 mJ/mm^2^, which is generally well tolerated by soft tissue and bone^24^. This energy range has been safely used in clinical practice to treat various musculoskeletal conditions with minimal side effects^13,25–27^.

Another difference lies in the methodology of cell counting: Wanner et al. exclusively recorded the surviving bacteria directly from the stainless steel surface using colony-forming units (CFU). In contrast, in our study the number of bacteria in the surrounding solution was determined—in addition to CFU counts, also using an XTT assay to quantify biofilm dissolution and cell viability in a differentiated manner. While Wanner et al. demonstrated that low-energy shock waves can increase the antibiotic sensitivity of biofilms, our study shows that low-energy ESW alone already exerts a biofilm-disrupting effect and detach individual bacterial cells from the biofilm matrix^22^. The disruption of biofilms by low-energy shock waves may be partly due to biophysical changes in bacterial membranes. Molecular dynamics simulations have demonstrated that shock wave propagation can induce structural deformation in lipid bilayers and facilitate water penetration. This leads to transient increases in membrane permeability^28^. This mechanism could explain the observed detachment of viable bacteria from the biofilm matrix without loss of metabolic activity, as reflected in the XTT and CFU results.

Similar findings were also observed in a recent study by Wu et al., which investigated the influence of low-energy ESW (0.12 mJ/mm^2^, 100 or 300 impulses) on uropathogenic *Escherichia coli* both in vitro (bacterial suspension) and in a rat model of cystitis. The authors demonstrated that ESW altered bacterial cell membrane gene expression and increased the antibiotic sensitivity of *E. coli*. Furthermore, they observed a reduction in bacterial load and attenuation of the inflammatory response in the bladder^29^.

However, a separate study, which also focused on low-energy ESW, yielded controversial results. Madron et al. were unable to detect any significant bactericidal effect of low-energy shock waves (0.15 mJ/mm^2^, up to 2000 impulses) on biofilms of *Escherichia coli* and *Staphylococcus epidermidis* on cortical bone screws. This discrepancy may be attributable to differences in bacterial strains, biofilm growth conditions, or surface materials. Furthermore, the 18-h biofilm formation differs significantly from the 48-h cultivation used in this study. Additionally, there is a discrepancy in the CFU measurement. In the study by Madron et al., the CFU count was performed after sonication of the screws with diluting and plating, whereas in this study, we directly detected the detachment of individual cells^30^.

In summary, low-energy extracorporeal shock waves have the potential to affect the matrix of *Staphylococcus aureus* biofilms on polyethylene and release cells into the surrounding solution. These findings offer promising approaches for improving the diagnosis of periprosthetic infections. However, further research is required to optimize their clinical application. In a clinical scenario, shock wave application could be followed by a minimally invasive joint aspiration to collect dislodged bacteria. The retrieved synovial fluid could then be analyzed via standard microbiological culture and analysis for white blood cell count, polymorphonuclear percentage and other biomarkers such as alpha-defensin or leukocyte esterase^31,32^, improving pathogen identification even in culture-negative PJI cases. The principle is analogous to implant sonication after implant removal, which increases diagnostic sensitivity by dislodging adherent bacteria from the prosthesis into the diagnostic sample^9,11^. As a less invasive alternative to implant retrieval, this approach could serve as a valuable adjunct to current diagnostic strategies for suspected periprosthetic joint infection. However, methodological differences between the studies indicate that standardisation of parameters and further in vivo investigations are required to validate the clinical relevance and to comprehensively investigate potential synergies between ESW and antimicrobials.

Beyond diagnostics, the treatment of PPIs also plays a major role. In this regard, a combined approach with antibiotics, as proposed by Wanner et al.^22^, could be integrated into future studies to harness potential synergistic effects and enhance clinical applicability. These mechanisms may be particularly relevant in persistent biofilm infections, as they could improve antibiotic penetration and efficacy. In addition, the results of Wu et al. support this assumption, demonstrating that low-energy ESW can influence bacterial cell membranes and modify their gene expression profiles^29^. Furthermore, the findings of Wanner et al. suggest that combining shock waves with antimicrobial substances may not only enhance their synergistic effects^22^, but also reduce the required antibiotic dose, potentially minimizing the risk of side effects.

## Limitations

The main limitation of the present study is that it is an in vitro experiment, which restricts the transferability of the results to clinical situations. The conditions created in the laboratory cannot fully simulate the complex physiological conditions in the human body. In particular, the interactions between biofilm, implant material and the immune system play a decisive role in vivo. Moreover, important anatomical structures such as skin layers, subcutaneous tissue and the joint capsule are absent in this in vitro experiment. In a clinical situation, these factors could potentially reduce the penetration depth of the shock waves, thereby diminishing energy transfer to the biofilm.

Further in vivo studies are therefore essential to validate the efficacy and safety of shockwave therapy in clinical application. While CFU and XTT assays provided quantitative information about bacterial detachment and viability, future studies should include structural analyses such as scanning electron microscopy (SEM) or confocal laser scanning microscopy (CLSM) to characterize morphological changes in the biofilm matrix. It would also be beneficial to investigate additional relevant bacterial strains and different implant materials to better evaluate the general applicability and potential uses of the method. Given the variability in biofilm composition, architecture, and response to mechanical disruption between bacterial species and material surfaces, generalization to other organisms or substrates must be made with caution. Moreover, thermal tests could also be carried out, as it is known that an increase in temperature can have an influence on the integrity of the biofilm^33^. During the tests, sample fluid is also removed so there was minimal loss of liquid around the patella. However, as only approximately 12% of the total volume was extracted, leaving about 18 ml of phosphate-buffered saline (PBS), the thickening effect of the remaining liquid is negligible and can therefore be considered negligible. This minor loss has no significant influence on the results of the tests.

## Conclusion

This study demonstrates that shock waves with an energy level of 0.13 mJ/mm^2^ effectively disrupt *Staphylococcus aureus* biofilms on polyethylene surfaces. These results highlight the potential of low-energy shock wave therapy as a promising, non-invasive method for biofilm destabilization. However, further in vivo studies are necessary to validate these findings and assess the clinical applicability of this approach. If confirmed, low-energy shock wave therapy could not only enhance the diagnosis of periprosthetic infections when combined with joint puncture but also serve as a valuable adjunct therapy to enhance antibiotic efficacy or even offer a valuable implant-preserving treatment option for mature biofilms, thereby improving the overall management of periprosthetic infections.

## Data Availability

The datasets used and/or analyzed during the current study are available from the corresponding author on reasonable request.
